# Three-dimensional nanostructured substrates enable dynamic detection of ALK-rearrangement in circulating tumor cells from treatment-naive patients with stage III/IV lung adenocarcinoma

**DOI:** 10.1186/s12967-019-1779-5

**Published:** 2019-01-18

**Authors:** Weiling He, Di Xu, Zhuo Wang, Hui Wu, Xianhong Xiang, Bing Tang, Wenting Jiang, Yongmei Cui, Han Wang, Neng Jiang, Yu Sun, Yangshan Chen, Shuhua Li, Minzhi Hou, Yang Zhang, Liantang Wang, Zun-fu Ke

**Affiliations:** 1 Department of Pathology, The First Affiliated Hospital, Sun Yat-sen University, 58 Zhongshan Road II, Guangzhou, 510080 Guangdong People’s Republic of China; 2 Department of Gastrointestinal Surgery, The First Affiliated Hospital, Sun Yat-sen University, Guangzhou, 510080 Guangdong People’s Republic of China; 3 Department of Thoracic Surgery, The Central Hospital of Wuhan, No.26 Shenli Street, Jiang’an District, Wuhan, 430014 Hubei China; 4 Department of Interventional Radiology, the First Affiliated Hospital, Sun Yat-sen University, Guangzhou, 510080 Guangdong People’s Republic of China; 5 Department of Gyneacology, Sun Yat-sen Memorial Hospital, Sun Yat-sen University, Guangzhou, 510120 Guangdong People’s Republic of China; 6 Biomedical Engineering, University of Texas at El Paso, 500 West University Avenue, El Paso, TX 79968 USA

**Keywords:** Nanomaterials, Microfluidics, Circulating tumor cell, Lung cancer, Diagnostics

## Abstract

**Background:**

Circulating tumor cells (CTC) shows great prospect to realize precision medicine in cancer patients.

**Methods:**

We developed the NanoVelcro Chip integrating three functional mechanisms. NanoVelcro CTC capture efficiency was tested in stage III or IV lung adenocarcinoma. Further, ALK-rearrangement status was examined through fluorescent in situ hybridization in CTCs enriched by NanoVelcro.

**Results:**

NanoVelcro system showed higher CTC-capture efficiency than CellSearch in stage III or IV lung adenocarcinoma. CTC counts obtained by both methods were positively correlated (r = 0.45, p < 0.05). Further, Correlation between CTC counts and pTNM stage determined by NanoVelcro was more significant than that determined by CellSearch (p < 0.001 VS p = 0.029). All ALK-positive patients had 3 or more ALK-rearranged CTC per ml of blood. Less than 3 ALK-rearranged CTC was detected in ALK-negative patients. NanoVelcro can detect the ALK–rearranged status with consistent sensitivity and specificity compared to biopsy test. Furthermore, the ALK-rearranged CTC ratio correlated to the pTNM stage in ALK-positive patients. Following up showed that CTCs counting by NanoVelcro was more stable and reliable in evaluating the efficacy of Clozotinib both in the short and long run compared with CellSearch. Changing of NanoVlecro CTC counts could accurately reflect disease progression.

**Conclusion:**

NanoVelcro provides a sensitive method for CTC counts and characterization in advanced NSCLC. ALK-rearrangement can be detected in CTCs collected from advanced NSCLC patients by NanoVelcro, facilitating diagnostic test and prognosis analysis, most importantly offering one noninvasive method for real-time monitoring of treatment reaction.

**Electronic supplementary material:**

The online version of this article (10.1186/s12967-019-1779-5) contains supplementary material, which is available to authorized users.

## Background

Circulating tumor cells (CTC) are cancer cells that exist in the circulation system of patients with solid cancers, after detaching from primary tumor lesions. It is said that CTC holds great promise in tailoring treatment decision for cancer patients [1]. CellSearch system as the most classic CTC enrichment system based on detection of tumor cells that express epithelial cell adhesion molecule (EpCAM), was effective to predict prognosis of many cancers, especially breast and lung cancers [1–3]. Furthermore, it is the only method approved by the US Food and Drug Administration (FDA) for the enumeration of CTC in certain advanced cancers. Detection of 5 CTCs per 7.5 ml or higher can predict prognosis regardless of disease histology and molecular subtype, type of treatment, or whether the patient had recurrent or de-novo metastatic disease [4].

Over the past years, a diverse suite of techniques including biophysical (e.g., density gradient centrifugation devices, size-based filtration systems, cell dielectric properties-based isolation assays, microfluidics-based technologies, etc.) and biochemical (e.g., capture-agent-labeled immunomagnetic beads, CTCs-chip and nanoparticle enrichment devices, etc.) methodology-based enrichment assays by their different properties had been demonstrated [5, 6]. One such most widely-used CTC assay CellSearch mentioned above is based on the technique of capture-agent-coated magnetic beads. More recently, nanostructured cell-affinity substrate coated with cancer-cell capture agents (e.g., epithelial cell adhesion molecule antibody, anti-EpCAM) has also been applied to enrich and immobilize CTCs, exhibiting improved cell-capture efficiency [7–13]. However, the overall enrichment efficacy is still discontented by use of these techniques.

Previously, we pioneered a NanoVelcro Chip owning anti-EpCAM [14, 15] coated 3D-nanostructured substrate [16], showed enhanced CTCs enrichment efficacy compared to CellSearch [17] in prostate cancer. Our NanoVelcro Chip integrates three functional features: (1) anti-EpCAM coating for capturing EpCAM-positive cells through immunological reaction, (2) a silicon nanopillar (SiNP) substrate with special microstructured surfaces, which exhibits improved cell-capture efficiency owing to enhanced local topographic interactions [18] between the SiNP substrates and nanoscale cellular surface components [17], (3) an overlaid polydimethylsiloxane (PDMS) chip with a serpentine chaotic mixing channel [8, 19–21] forming special microfluidic that increased cell-substrate contact frequency. Despite the enhanced CTCs capture ability, the controlled study did not perform in lung cancer and the subsequent CTC cellular characterization (e.g., driver oncogene mutation status) consistency which is of great clinical significance for therapy guidance between the two methods weren’t further analyzed in this study.

As the most common sensitive gene mutations for lung cancer, a fusion gene between the anaplastic lymphoma kinase (ALK) gene and echinoderm microtubule associated protein-like 4 (EML4) was identified in 3% to 7% of unselected NSCLC patients [22], and further proved to be a potent oncogenic driver [23, 24]. Crizotinib as an ALK inhibitor [9, 24], was approved by the FDA for treating advanced ALK-positive NSCLC patients [10] and showed good therapeutic effect. Recently, the Vysis ALK Break-Apart FISH Probe Kit (Abbott Molecular, Inc.) was approved to detect ALK rearrangement status on small biopsies or fine-needle aspirated tissue. Considering the biopsy risk, medical cost and patient acceptance, it is difficult to repeatedly obtain tumor specimens, which are imperative in determining the therapeutic effect, exploring predictive biomarkers and choosing alternative treatment timely. In patients with advanced NSCLC, tumor specimen is more difficult to obtain because surgery is rarely a component of treatment [11] in advanced lung cancer patients. Thus, developing a noninvasive and effective assessment platform, that could predict whether patients can benefit from primary treatment and diagnosis if an ALK-rearrangement presents identifying NSCLC patients benefiting from Crizotinib treatment, is imperative and invaluable.

Based on the predicament faced above, our present study was to determine if NanoVelcro platform could capture CTC from the advanced NSCLC patient blood with improved efficiency compared to CellSearch system by utilizing similar enrichment technique. Moreover, the second endpoint of our study was to determine whether an ALK-rearrangement could be detected in CTCs enriched by NanoVelcro assay from ALK-positive advanced NSCLC patients, facilitating treatment strategy design and prognosis monitoring.

## Materials and methods

### Patients

**Fig. 1:**
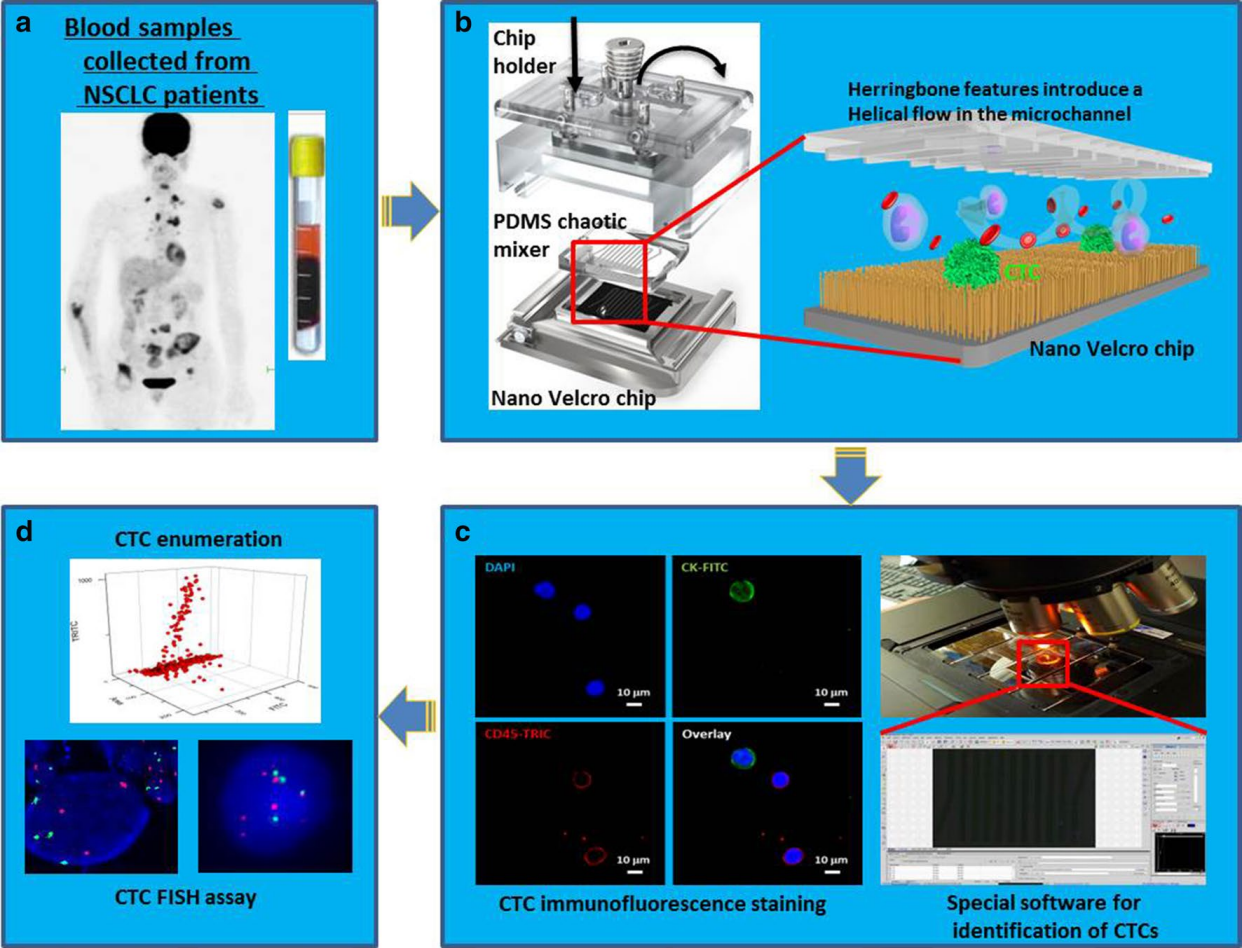
**a** 1.0 mL of blood is collected from NSCLC patients. **b** CTCs are captured in Nano Velcro chip. **c** Immunostaining assay is carried out to identify CTCs. **d** FISH assay is used to detect the ALK status in CTCs

This study was approved by the institutional ethics committee of the First Affiliated Hospital of Sun Yat-sen University, China (A-084). Totally forty-one lung cancer patients were enrolled in our study in which twenty-one patients with positive ALK-rearranged status in tumor tissues were set as observation group and twenty patients with negative ALK-rearranged status were set as control, and consistent consent to publish from all the participants had obtained. All the forty-one patients were with stage III or IV lung adenocarcinoma. Peripheral blood samples were collected from all the patients before initiating standard treatment. In the follow up study, samples were collected from one patient undergoing Crizotinib treatment for 8 months. All blood samples (1 ml per person) were collected into EDTA-containing vacutainer tubes (BD bioscience) and then proceed to further procedures (Fig. 1).

### NanoVelcro chip fabrication

**Fig. 2:**
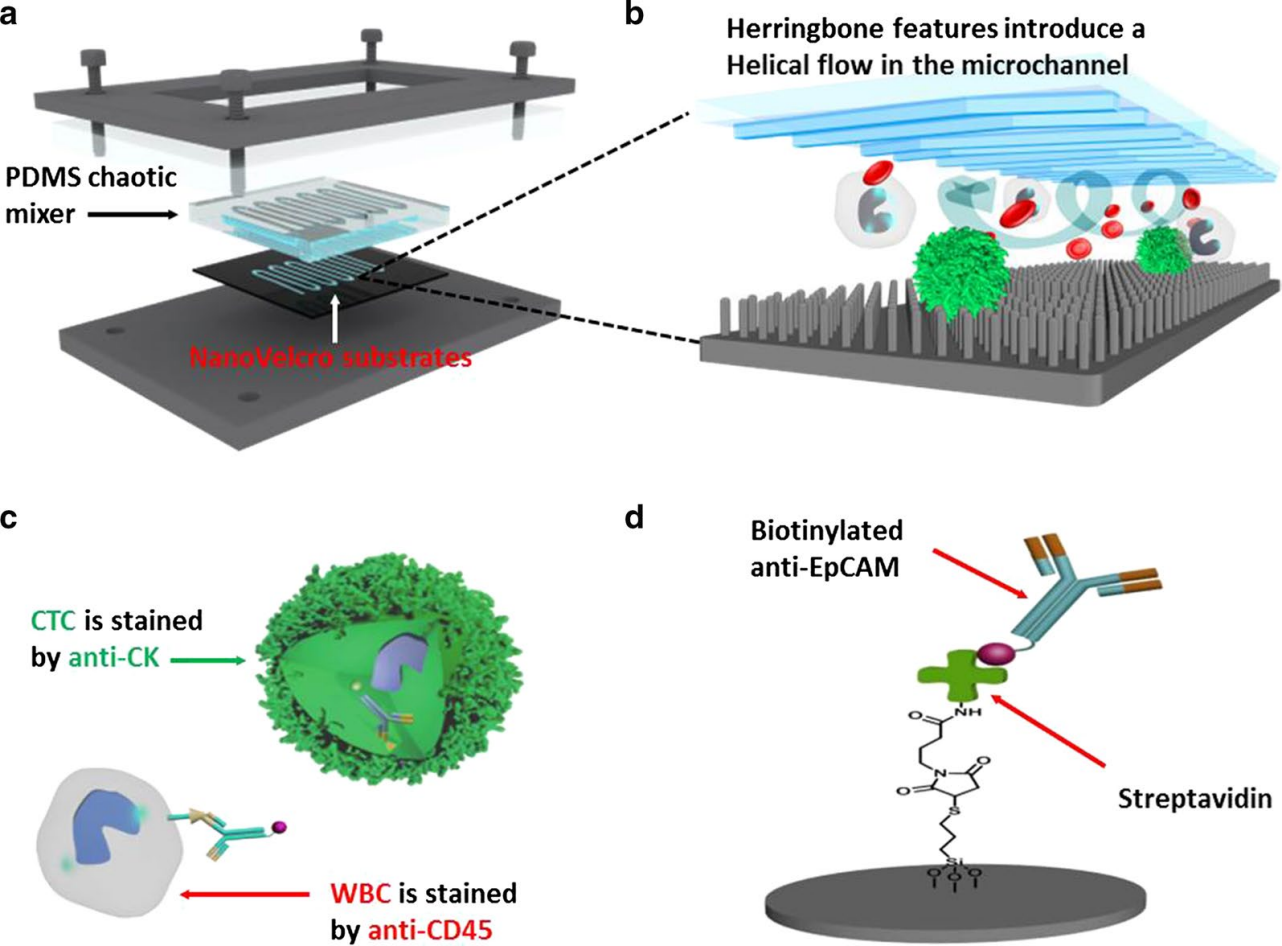
NanoVelcro device configuration and work mechanism. **a** NanoVelcro CTC Chip is composed of an overlaid PDMS-based chaotic mixer, a patterned silicon nanowire (SiNW) substrate, and a multilayer chip holder to assemble both functional components together. **b** Herringbone features introduce a helical flow in the microchannel facilitating the immunological recognition between CTC and anti-EpCAM coated SiNW substrate. **c** Schematic representation of an immunofluorescence protocol developed for identification of CTC (CK+/CD45−/DAPI+) from non-specifically captured WBCs (CK−/CD45+/DAPI+) and cell debris. **d** Silanation reaction were employed to covalently link streptavidin onto the SiNW substrate, allowing conjugation of biotinylated anti-EpCAM prior to CTC detection studies

NanoVelcro CTC chip is composed of three parts: (1) a serpentine chaotic mixer chip made of polydimethylsiloxane (PDMS), (2) a patterned silicon nanowire (SiNW) substrate with high-affinity anti-EpCAM coating, and (3) a home-machined holder set to sandwich a well-aligned PDMS mixer chip with the SiNW substrate (Fig. 2). The PDMS mixer chips were fabricated using a standard soft-lithography method. Consistent channel structure and PDMS thickness were achieved using this method. The fabrication method for the SiNW substrate was reported earlier [7, 17]. After DI water rinsing and nitrogen blow-dry, the substrate was ready for subsequent streptavidin coating. Streptavidin coating was also conducted following a previously established protocol.

Prior to each test, the PDMS chaotic mixer chip and streptavidin-coated substrate were sandwiched together using a home-machined holder set consisting of (1) one stainless steel bottom plate, (2) one poly methyl methacrylate (PMMA) clapboard, (3) one stainless steel top frame, and (iv) a screw at each corner (Fig. 1a). Finally, the top frame was anchored to the PMMA clapboard and bottom plate by screws to form a constant pressure seal to prevent leakage. The anti-EpCAM solution was then loaded into the channel by a syringe pump (KDS 200, KD scientific) for conjugation. Before sample injection, the channel required multiple PBS 1× washings to remove free anti-EpCAM. This may dramatically shorten the time spent on whole chip scanning while reducing total reagent consumption for each test.

### FISH assay on tumor tissue

The dual-color FISH assay using the Vysis LSI *ALK* Break Apart Rearrangement Probe Kit (Abbott Molecular) is completed following procedure instruction. The Vysis *ALK* Break Apart FISH Probe Kit consists of two probes adjacent to the 3′ (red) and 5′ (green) ends of ALK. In cells with a native ALK status, the overlapping of probes results in a fused (3′ 5′, yellow) signal. The two characteristic ALK-rearrangement split patterns are the split of the 3′ (red) and 5′ (green) probes (a distance of more than two signal diameters was considered a split) or an isolated single or amplified 3′ (red) signal. Signals were enumerated in at least 100 tumor nuclei, and FISH-positive cases were defined as those with more than 15% split or isolated signals [11, 25].

### CTC enrichment by CellSearch and NanoVelcro

CTC enrichment by NanoVelcro was performed on 1.0 ml of blood similar to previous report [7, 17]. CTC were enumerated by using CellSearch (Veridex) from 7.5 ml of blood as previously described [11, 25]. In order to visualize captured cells on SiNW substrate, additional steps of immunocytochemistry were employed by loading the microchannel with 100 μl of fluorophore-labeled antibody [anti-TTF-1 (Thyroid transcription factor 1), anti-CEA (carcinoembryonic antigen), anti-CK-7 (cytokeratin-7) and p63 (Dako, Glostrup, Denmark)] solution (20 μl/1 ml initial concentration) for overnight incubation at 4 °C after the step of cell permeabilization. After image acquisition, CTCs were identified by combination of a series of criteria: positive staining of anti-cytokeratin (PE) and DAPI; negative staining of anti-CD45 (FITC); and characteristic phenotypes and morphology of cancer cells. The cells with phenotypes of positive anti-CD45 (FITC) and DAPI, but negative anti-cytokeratin (PE) were excluded as lymphocytes. Experimental approaches used to characterize CTC collecting from NanoVelcro are detailed in Additional file 1.

### FISH assay of NanoVelcro enriched CTC

Each step of the FISH method was optimized for highest cell recovery. Hybridization was performed by using the Vysis LSI Dual Color *ALK* Break Apart Rearrangement Probe Kit [Abbott Molecular, (37 °C, pepsin (Sigma, USA) at 10% in an HCL 0.01 N)]. The ALK status was validated blindingly by an experienced cytogenetician. FISH signals were analysed either manually using an epi-fluorescence microscope (Nikon), or an automated scanning Ariol system with a Leica DM6000 microscope (Leica Microsystems, Mannheim, Germany). Cells were detected by magnification of 1000× for a manual scan or by magnification of 63× for an automatic scan. FISH procedures were pre-established using serial dilutions of ALK-rearranged H2228 cells spiked into peripheral blood samples of healthy individuals.

### Statistical analysis

Paired sample test was used to compare the CTC counts between CellSearch and NanoVelcro. Linear regression plots were computed for CTC counts obtained by CellSearch and NanoVelcro techniques. Their concordance rate was calculated by Pearson test. In order to differentiate ALK-positive and ALK-negative patients, the optimal cutoff of ALK-rearranged CTC counts was evaluated using receiver operating characteristic (ROC) curve to maximize the sum of sensitivity plus specificity in terms of ALK status prediction. The Chi square analysis was used to evaluate whether ALK-rearrangement status in NanoVelcro captured CTC correlated with the ALK-rearrangement status in tumor biopsy. The Cohen’s kappa coefficient was used to assess the concordance between these two methods. The sensitivity, specificity, positive and negative predictive values of ALK-rearrangement CTC were calculated using FISH results in tumor sample as gold standard. Further, the concordance rate between conventional pTNM stage and CTC counts detected by NanoVelcro, pTNM stage and *ALK*-rearranged positive CTC ratio in ALK positive patients were calculated by Spearman test. Data presented as the mean ± standard deviation (SD). Statistical analyses were performed using SPSS 16.0 software (SPSS Inc. Chicago, IL). Differences were considered significant, if p value was less than 0.05.

## Results

### NanoVelcro with higher efficiency than CellSearch

**Fig. 3:**
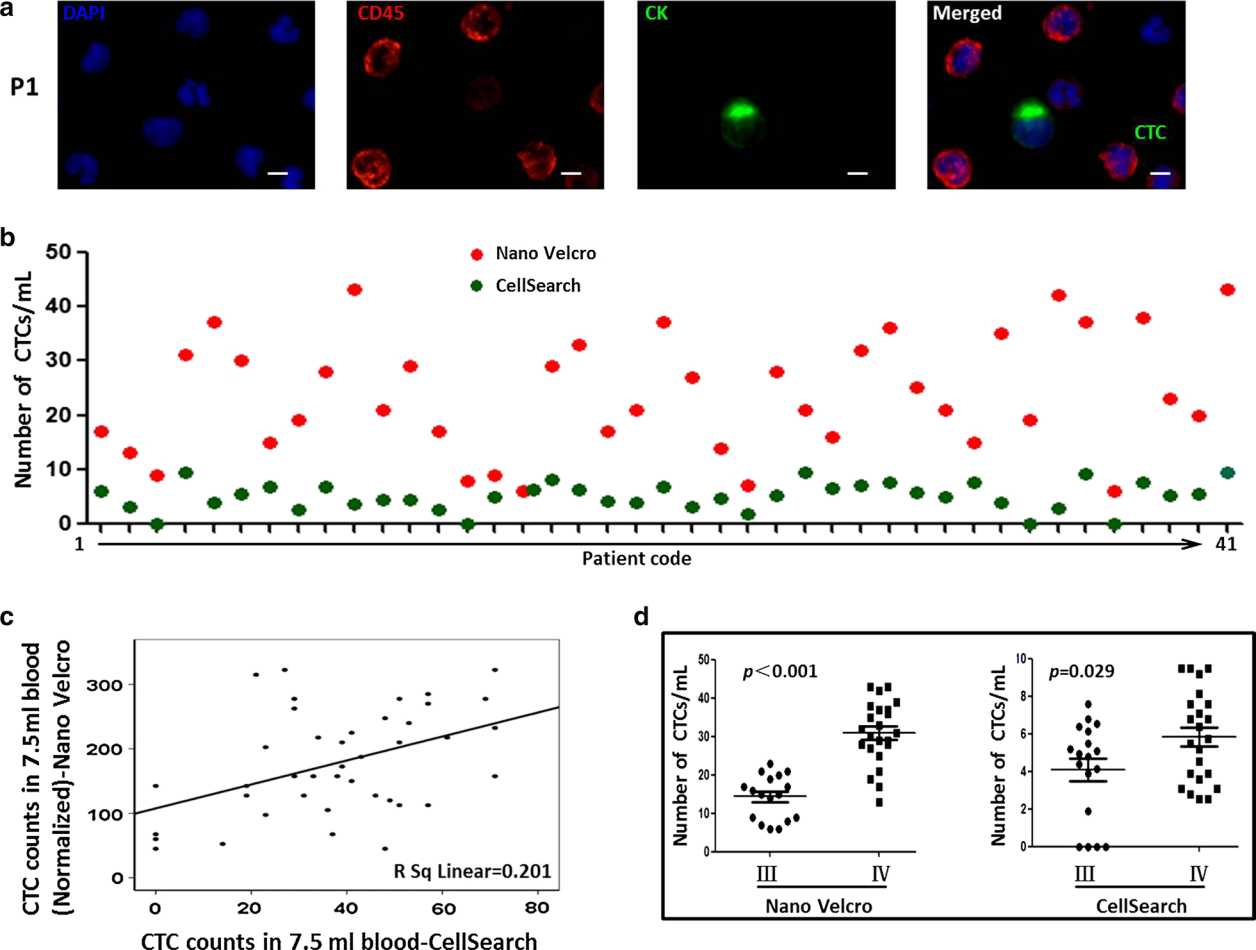
Comparison of NanoVelcro and CellSearch in CTC capture ability. **a** Immunofluorescence staining of CTC immobilized on NanoVelcro substrates. Typical micrographs of CTC and WBCs were immobilized on a NanoVelcro substrate. Scale: white bars correspond to 10 μm. **b** The enumerated CTC by NanoVelcro from 1.0 ml blood and by CellSearch from 7.5 ml in NSCLC adenocarcinoma patients at their first visit (Normalized to 1.0 ml scale for comparison). Red represents the enumerated CK+/CD45− CTC by NanoVelcro in patient blood; Green represents CellSearch counts CTC. **c** Linear regression plots were computed for CTC counts obtained by CellSearch and NanoVelcro (Normalized to 7.5 ml scale for comparison). **d** Correlation analysis between pTNM stage with NanoVelcro and CellSearch CTC counts respectively in all NSCLC adenocarcinoma patients (Normalized to 1.0 ml scale for comparison)

NanoVelcro can capture typical CTC in blood of advanced NSCLC patients, characterized as CD45-negative and CK-positive (Fig. 3a). In accordance with previously report [17], CTC counts were higher by NanoVelcro than CellSearch (178.17 ± 81.45 vs. 37.95 ± 19.54 per 7.5 ml, p < 0.05) (Fig. 3b). More importantly, CTC counts obtained with both methods were moderately correlated (r = 0.45, p < 0.05) (Fig. 3c).

CTC were detected in all advanced NSCLC ALK-positive and ALK-negative patients using NanoVelcro, meanwhile in 19 of 21 (90.49%) advanced NSCLC ALK-positive patients and 18 of 20 (90%) negative patients by CellSearch (Tables 1, 2). Mean values of 35.67 ± 18.02 and 167.50 ± 80.33 CTC per 7.5 ml were detected by CellSearch and NanoVelcro in ALK-positive patients, respectively (Table 1). Meanwhile, they detected 40.35 ± 21.22 and 189.38 ± 82.37 CTCs per 7.5 ml in ALK-negative patients respectively (Table 2). Further analysis showed that Correlation between CTC counts and pTNM stage determined by NanoVelcro was more significant than that determined by CellSearch (p < 0.001 vs p = 0.029, Fig. 3d).

**Table 1:** Numbers and Percentages of ALK-Rearranged Cells in Tumors and in CTC of ALK-Positive Patients

Patient	Sex	Age (y/o)	Smoking status (y/o)	Adenocarcinoma subtype	Clinical stage	Tumor		CTCs by NanoVelcro			CTC number by CellSearch (7.5 ml)
						Biopsy origin	% of rearranged cells	Rearranged CTCs (per ml)	% of rearranged CTCs	Total CTCs (per ml)	
P1	F	51	2	Papillary	III	Pleura (MS)	43	6	35	17	46
P2	F	47	0	Mucinous	IV	Lung (PT)	38	5	39	13	23
P3	M	43	0	Papillary	III	Node (MS)	27	3	22	9	0
P4	M	68	15	Mucinous	IV	Pleura (MS)	57	27	87	31	71
P5	M	54	21	Papillary	IV	Bone (MS)	96	32	87	37	29
P6	M	69	41	Solid	IV	Node (MS)	86	21	70	30	41
P7	F	37	0	Mucinous	III	Node (MS)	41	7	47	15	51
P8	F	58	0	Solid	IV	Lung (PT)	47	17	90	19	19
P9	F	57	0	Solid	IV	Lung (PT)	37	21	75	28	51
P10	F	63	0	Mucinous	IV	Lung (PT)	82	36	84	43	27
P11	M	71	10	Mucinou	III	Pleura (MS)	31	9	43	21	33
P12	M	41	0	Mucinou	IV	Node (MS)	71	25	86	29	34
P13	F	82	0	Mucinou	IV	Brain (MS)	51	17	100	17	19
P14	F	35	0	Solid	III	Lung (PT)	19	3	33	8	0
P15	F	47	0	Solid	III	Lung (PT)	47	9	100	9	37
P16	M	49	0	Papillary	III	Node (MS)	49	3	50	6	48
P17	F	53	12	Mucinou	IV	Node (MS)	59	24	83	29	61
P18	F	49	1	Solid	IV	Lung (PT)	42	19	58	33	48
P19	F	67	0	Mucinou	III	Lung (PT)	36	8	47	17	31
P20	M	69	19	Solid	III	Node (MS)	38	10	48	21	29
P21	M	45	0	Solid	IV	Lung (PT)	79	28	76	37	51

*ALK* anaplastic lymphoma kinase, *CTC* circulating tumor cell, *F* female, *M* male, *MS* metastatic site, *PT* primary tumor

**Table 2:** Numbers and percentages of ALK-rearranged cells in tumor and in CTC of ALK-negative patients

Patient	Sex	Age (y/o)	Smoking status (y/o)	Adenocarcinoma subtype	Clinical stage	Tumor		CTCs by NanoVelcro			CTC number by CellSearch (7.5 ml)
						Biopsy origin	% of rearranged cells	Rearranged CTCs (per ml)	% of rearranged CTCs	Total CTCs (per ml)	
PN1	M	39	10	Mucinous	IV	Bone (MS)	0	2	7	27	23
PN2	F	47	0	Solid	III	Lung (PT)	0	0	0	14	36
PN3	F	71	0	Papillary	III	Lung (PT)	0	0	0	7	14
PN4	F	52	0	Solid	IV	Pleura (MS)	0	0	0	28	39
PN5	M	67	30	Papillary	IV	Node (MS)	0	1	5	21	71
PN6	F	54	4	Solid	III	Node (MS)	0	1	6	16	49
PN7	M	45	0	Mucinous	IV	Brain (MS)	0	0	0	32	53
PN8	M	37	0	Solid	IV	Lung (PT)	0	2	6	36	57
PN9	F	75	0	Solid	IV	Pleura (MS)	N/A	0	0	25	43
PN10	F	58	0	Mucinous	III	Lung (PT)	0	0	0	21	38
PN11	F	63	0	Solid	III	Pleura (MS)	0	0	0	15	57
PN12	M	54	27	Mucinou	IV	Node (MS)	0	1	3	35	29
PN13	M	77	0	Solid	III	Lung (PT)	0	0	0	19	0
PN14	F	39	3	Solid	IV	Node (MS)	0	0	0	42	21
PN15	F	53	0	Solid	IV	Bone (MS)	0	2	5	37	69
PN16	F	61	0	Mucinous	III	Lung (PT)	0	0	0	6	0
PN17	M	60	0	Papillary	IV	Node (MS)	0	0	0	38	57
PN18	M	71	47	Solid	III	Lung (PT)	0	0	0	23	39
PN19	F	47	0	Mucinou	III	Lung (PT)	0	0	0	20	41
PN20	F	63	0	Papillary	IV	Bone (MS)	0	0	0	43	71

*ALK* anaplastic lymphoma kinase, *CTC* circulating tumor cell, *M* male, *MS* metastatic site, *N/A* not available, *PT* primary tumor

### Detecting ALK-rearrangement in CTC captured by NanoVelcro

**Fig. 4:**
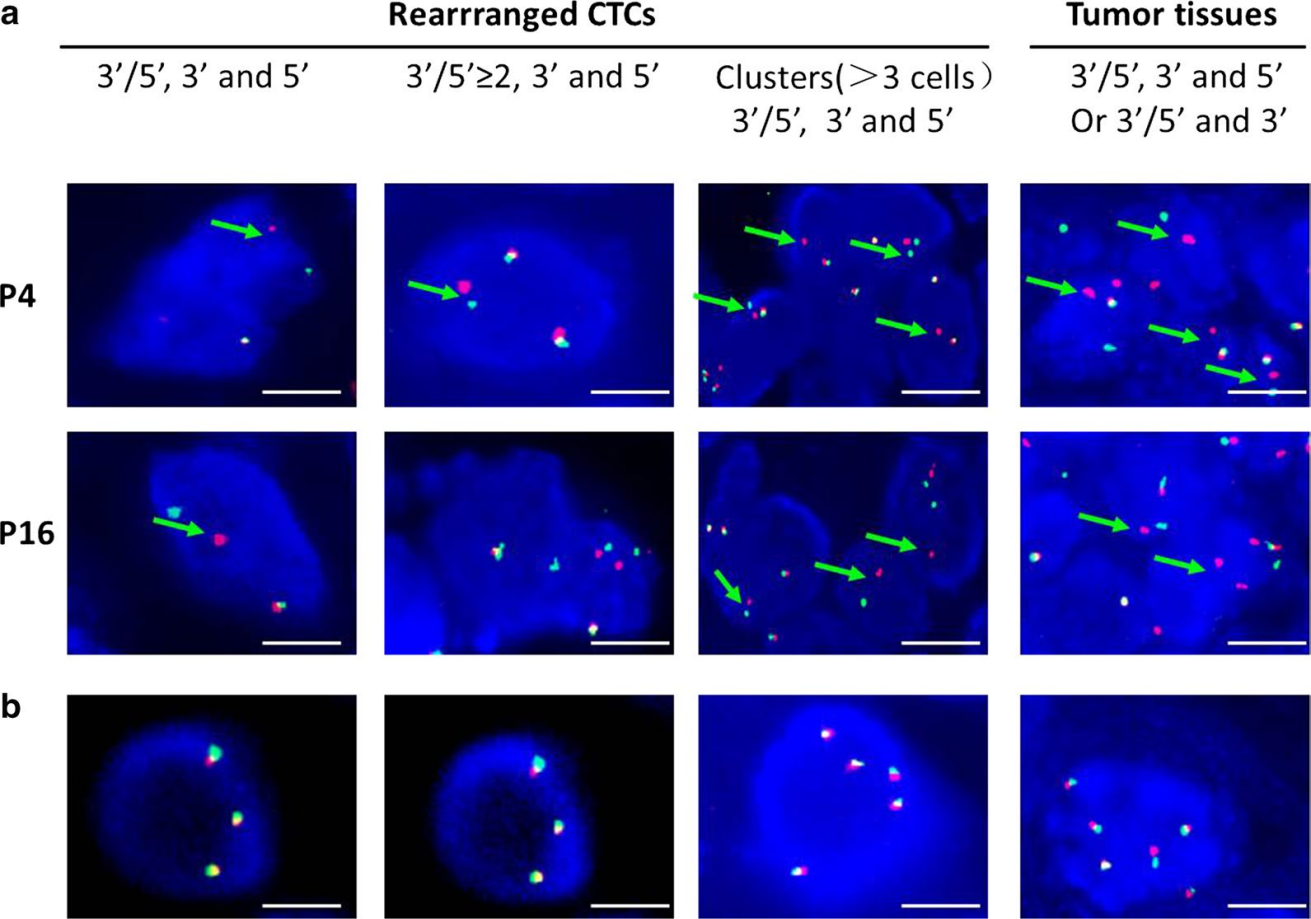
Detection of anaplastic lymphoma kinase (ALK) gene abnormalities in circulating tumor cells (CTC) and tumor specimens of ALK-positive patients. **a** Examples of isolated or clusters of ALK-rearranged CTCs detected by filter-adapted fluorescent in situ hybridization (FISH) and of ALK-rearranged tumor cells in tumor specimens detected by FISH. Green arrows show an ALK rearrangement with a split 3′ and 5′ (red/green) signal. Red arrows show an ALK rearrangement with only the 3′ signal. **b** Examples of isolated CTCs with a gain of native ALK copies. In cells with a native ALK status, the overlapping of probes results in a fused (3′ 5′, yellow) signal. Scale: white bars correspond to 10 μm

All patients were determined by an *ALK*-rearrangement FISH Probe Kit in biopsies of primary tumors or metastases. ALK-positive patients were defined as those with more than 15% split or isolated signals (Fig. 4). Twenty-one patients presented ALK-positive status with the percentage of ALK-rearranged cells ranging from 19 to 96% (Table 1). All 21 ALK-negative patients were examined with a negative FISH result (Table 2).

**Fig. 5:**
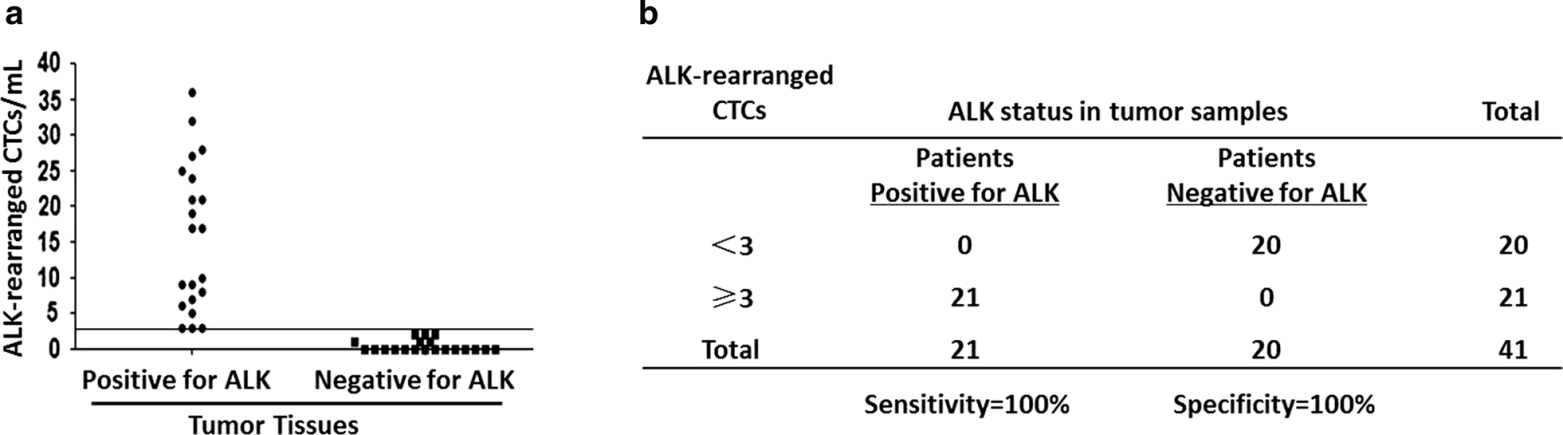
Determination of the anaplastic lymphoma kinase (ALK)–rearranged circulating tumor cells (CTC) optimal cutoff value in ALK-positive and ALK negative patients. **a** Prevalence of ALK-rearranged CTC in ALK-positive and ALK-negative patients. **b** Determination of the cutoff value for 3 ALK-rearranged CTC per ml blood by using receiver operating characteristic curve analysis. *NPV* negative predictive value, *PPV* positive predictive value

**Fig. 6:**
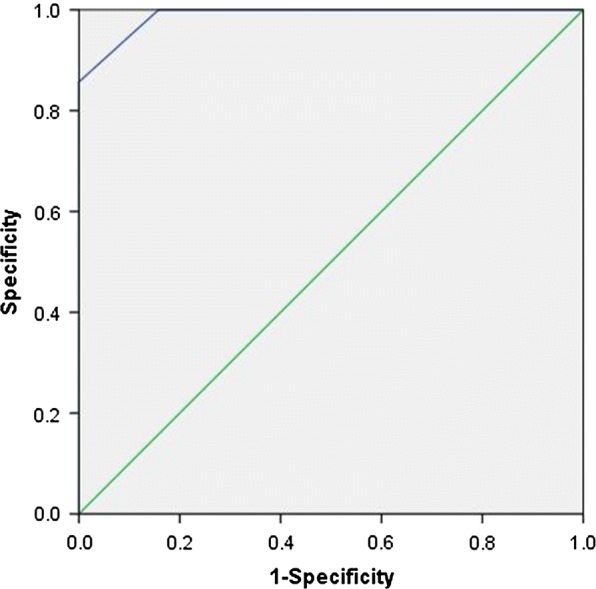
Receiver operating characteristic (ROC) curve in differentiating ALK-positive and ALK-negative patients, the corresponding AUC was approximately 0.989

By NanoVelcro, CTC harboring an ALK-rearrangement were detected in all ALK-positive patients with a mean value of 15.71 ± 10.33 CTC per ml (Table 1). All ALK-positive patients had 3 or more ALK-rearranged CTCs per ml of blood (Fig. 5a). No or only 2 ALK-rearranged CTC was detected in blood of the 20 patients with ALK-negative NSCLC (mean, 0.45 CTC per ml; Table 2). Receiver operating characteristic curve analysis indicated that a cutoff value of 3 ALK-rearranged CTC per ml blood had a sensitivity of 100% and a specificity of 100% (Fig. 5a). The concordance between FISH based on NanoVelcro captured-CTC and tumor specimen quantified by the κ coefficient was 99.99% (Fig. 5b), area under ROC curve was approximately 0.989 (Fig. 6). Mean percentages of ALK-rearranged CTC were 64.76 ± 24.03% in ALK-positive patients and were 1.6% in ALK-negative patients (Tables 1, 2).

In order to differentiate ALK-positive and ALK-negative patients, the optimal cutoff of ALK-rearranged CTC counts was evaluated using receiver operating characteristic (ROC) curve to maximize the sum of sensitivity plus specificity in terms of ALK status prediction.

### ALK-rearranged status of CTC aid treatment strategy decision

**Fig. 7:**
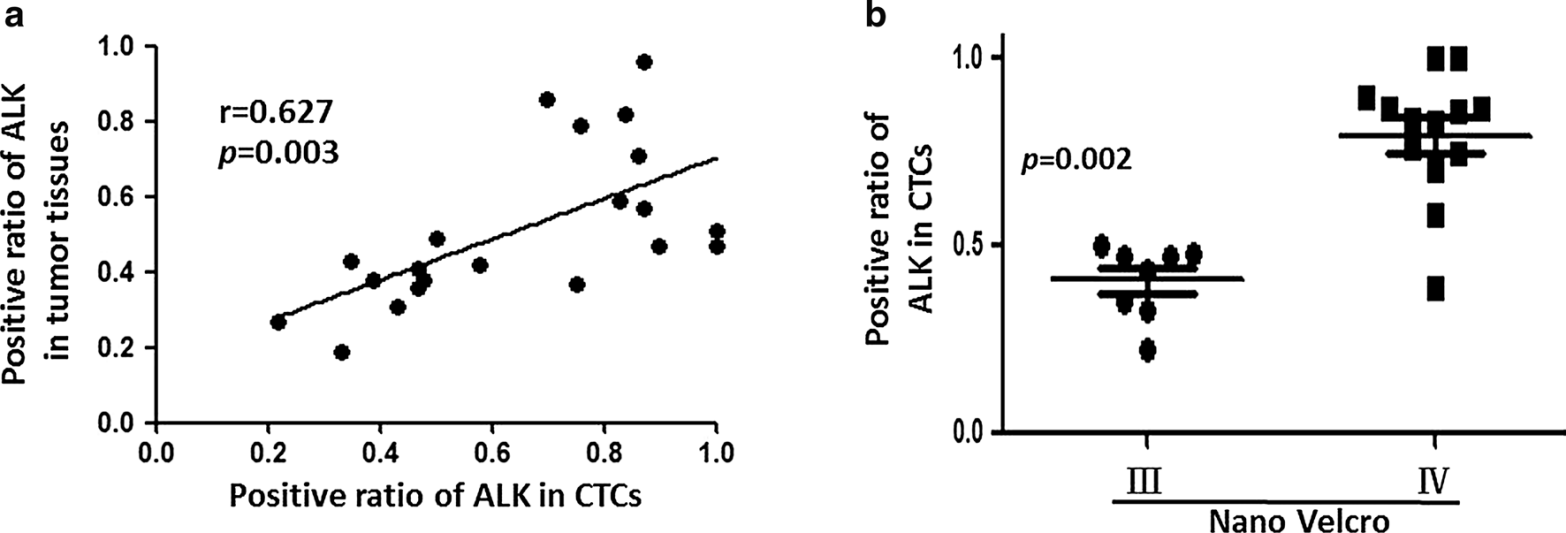
Clinical significance of circulating tumor cells (CTC) harboring anaplastic lymphoma kinase (ALK) in ALK-positive patients. **a** CTC counts by NanoVelcro positively correlated to CellSearch technique in ALK-positive patients. **b** In ALK-positive patients, CTC counts by NanoVelcro related to pTNM stage, but CellSearch

Detection and genomic analysis of CTC is anticipated to facilitate precision medicine as a potential real-time and noninvasive method in replace of invasive biopsy. Traditionally, the ALK mutation can only be tested in the tumor biopsy, which is very difficult to obtain under certain circumstance. This study offered one alternative method to identify ALK mutations from CTC isolated by NanoVelcro. Current strategies for isolating CTC are limited to complex analytic approaches that generate low yield and purity. In this study, ALK-rearrangement CTC was consistently detected in all patients harboring this mutation confirmed by the tumor specimen. In 21 ALK-positive patients, the ratio of ALK-rearrangement CTC positively correlated to the ratio of ALK positive cells in tumor tissue (Fig. 7a). This assay for detecting ALK mutations in advanced NSCLC patients through CTC captured by NanoVelcroC may be applied to screen ALK positive patient eligible for ALK-targeted treatment.

### Dynamically monitoring NSCLC progression through counting CTC during Crizotinib Treatment

Under selective pressure of therapy intervention, resistance is inevitable in anti-tumor strategy. Even patients treated with molecular targeted drugs will acquire resistance, commonly as a result of a secondary mutation. Thus, developing an effective method to monitor the treatment reaction and unveil the underlying resistance mechanism is invaluable. We demonstrated that CTC characterization owns prognostic value as well as the CTC counts. In ALK-positive patient, the ALK-rearrangement CTC ratio correlated to the pTNM stage (Fig. 7b), indicating that increased ALK-rearrangement CTC may predict advanced disease progression.

**Fig. 8:**
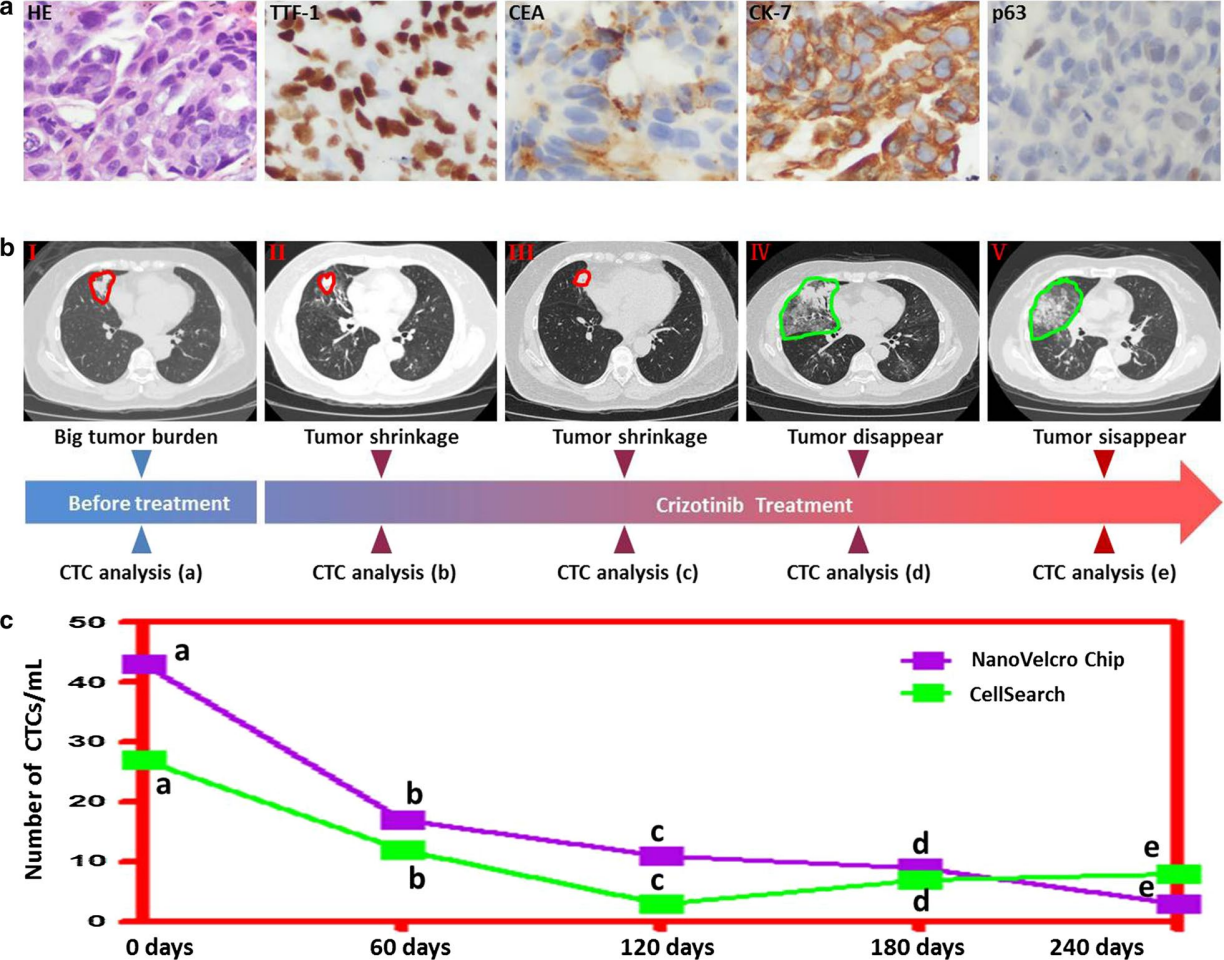
Dynamic change in circulating tumor cell (CTC) counts before and after initiation of crizotinib treatment for 8 months in one ALK-positive patient. **a** Tumor tissues from this patient were positive for TTF-1, CEA and CK-7. **b** Serial lung CT scan changes are presented. Lung tumor showed gradual shrinkage after crizotinib treatment. **c** Serial CTC counts by NanoVelcro and CellSearch are plotted along with radiological changes. CTC counts by NanoVelcro are always higher than CellSearch before lung tumor disappearing on CT scan after crizotinib treatment for 6 months. NanoVelcro accurately reflects the disease progressions with the disappearing of tumor, while CellSearch rebounded abnormally

Blood samples were collected from one patient for CTC enumeration by both methods during Crizotinib treatment for 8 months, meanwhile several times of radiological examination (CT scan) were performed according to the requirement of disease evaluation. Both NanoVelcro and CellSearch CTC counts initially dropped after the start of Crizotinib, accompanied by imaging relieve. All these changes keep in accordance to the improvement of clinical symptoms, indicating Crizotinib treatment effect. After treatment for 6 months, obvious tumor shrinkage presented in CT scan, while CTC counts of both methods dropped to the same level. However, in next 2 month, CTC counts from CellSearch rebounded abnormally disaccording with the tumor disappearing on CT scan. Meanwhile, NanoVlecro CTC counts presented further decrease, accurately reflecting disease progression (Fig. 8).

## Discussion

Our present study showed great improvement in CTC-capture efficiency with NanoVelcro chip compared with CellSearch in stage III or IV lung adenocarcinoma, which is in accordance with our previous findings [17], however the results need to be further validated by enlarging the sample size. Furthermore, CTC counts of the two methods positively correlated. Correlation between CTC counts and pTNM stage determined by NanoVelcro was more significant than that determined by CellSearch. CTC characterization analysis demonstrated that CTCs captured by NanoVelcro well reflect the ALK mutation status in tumor tissue, the ratio of ALK-rearrangement in CTCs positively correlated to that in tumor tissue. Furthermore, ALK-rearrangement CTC ratio detected by NanoVelcro correlated to the pTNM stage indicating that increased ALK-rearrangement CTCs may represent advanced disease status. Interestingly, as for therapeutic effect monitoring, in the short term (within the first 6 months), the two methods showed good consistency in evaluating the treatment efficacy of Clozotinib, but in the long run (the 8th month), compared with CellSearch, CTC counting by NanoVelcro was more stable and reliable in evaluating the efficacy of Clozotinib. Whilst, decreasing of NanoVlecro CTC counts could accurately reflect disease remission evaluated by chest CT image.

The ability to identify, isolate, propagate and characterize CTC subpopulations with noninvasive CTC-capture platform could deepen the understanding of blood metastasis mechanism, facilitate accurate evaluation of disease progression, continuously monitor the CTC (e.g. number, molecular and genetic characters) change to guide therapy. Although CellSearch is the only cell-affinity assay approved by the FDA and CTC numeration based on this platform and was proved to be an accurate prognostic indicator [1–3], whereas, it is gradually being depreciated due to “important limitations” in selecting CTC based on EpCAM which could lead to comparatively limited number of captured CTCs of epithe-lial cell phenotype only. However, metastasis begin with the epithe-lial–mesenchymal transition (EMT), in which tumor cells lose many of their ‘epithelial’ characteristics and become more like mesenchymal cells with the abil-ity to spread and invade tissue [26]. Thus, this kind of cell capture technique is likely to miss the cells of mesenchymal cell phenotype which is EpCAM-negative. To increase capture efficiency of CTCs, we developed this NanoVelcro platform detect CTC based on the similar immunological mechanism of CellSearch, but combined with special microstructured surfaces to strengthen topographic interactions and microfluidics to facilitate contact frequency. Microfluidics design may increase the contact between CTC and EpCAM coated substrate and intensify the immunological recognition affinity, maximizing the capture potential which CellSearch can’t accomplish. Expectedly, this platform identified more than several times of CTC than CellSearch in advanced NSCLC patient, showing improved capture efficiency. Additionally, since CellSearch may miss EpCAM-negative CTC, this drawback may be partially compensated by strengthened topographic interactions between CTC and NanoVelcro microstructured surfaces [27], this is also involved in our device by using special microstructured surfaces.

In our study, CTC characterization analysis demonstrated that ALK-rearrangement status in NanoVelcro captured CTCs showed reliable consistency with that in the tumor specimen, and ALK-rearrangement was consistently detected in CTC from lung cancer patients harboring this rearrangement. Traditionally, ALK mutation status can be only determined by tissue biopsy, which is invasive, expensive and time consuming. We demonstrated NanoVelcro platform that can non-invasively and accurately determine ALK mutations status in advanced NSCLC patients. Thus, NanoVelcro may be a surrogate to screen ALK positive patients eligible for ALK-targeted treatment, and CTCs captured by such technique and further FISH may resolve the problem of monitoring the genomic profiling of tumor, which may predict the treatment efficacy of Crizotinib and is hard to be achieved by traditionally tumor biopsy analysis. Furthermore, ALK-rearrangement CTC ratio detected by NanoVelcro correlated to pTNM stage indicating that increased ALK-rearrangement CTCs may represent advanced disease status.

Then we further demonstrate the ability to identify CTC number changes by NanoVelcro in response to Crizotinib therapies in one metastatic NSCLC patients. The results showed that, compared with CellSearch, CTCs counting by NanoVelcro was more stable and reliable in evaluating the efficacy of Clozotinib both in the short and long run. Whilst, changing of NanoVlecro CTC counts could accurately reflect disease progression. This might be due to the fact that the NanoVelcro assay can more accurately reflect the true number of CTCs. Our results are simillar to those reported [28, 29]. As the NanoVelcro generally presented much higher capture efficiency compared to the CellSearch™ assay, CTC counts by the NanoVelcro yield a wider dynamic range and may be a better predictor of disease progression [7]. Whilst, decreasing of NanoVlecro CTC counts could accurately reflect disease remission evaluated by chest CT image. Thus, NanoVelcro can be used to dynamically monitor the tumor morphology changes through analyzing CTC avoiding traditional tumor biopsy.

Briefly, NanoVelcro platform showed improved CTC-capture efficiency than CellSearch system, and the ALK-rearrangement status of captured CTCs kept in accordance with results from tumor specimen. This platform is primarily demonstrated to be an accurate, noninvasive and real-time detection of ALK mutations in treatment-naive patients with stage III or IV lung adenocarcinoma, optimizing the selection of ALK-inhibitor eligible patients and the dynamic monitoring of therapy reaction. Promisingly, Since NanoVelcro captured CTC are alive, these cells could potentially be expanded ex vivo for further molecular interrogation. Our future efforts will be devoted to preserve patient-derived CTC lines to select potential therapies options for individual cancer patients. We will also attempt to utilize NanoVelcro to predict the treatment reaction based on real-time monitoring the CTC counts and characterization changes in larger clinical cohorts to further confirm our findings.

## Conclusion

In this study, ALK-rearrangement can be detected in CTCs captured by Nano Velcro system, presenting high concordance with matched primary tumor tissues. Correlation between CTC counts and pTNM stage determined by NanoVelcro was more significant than that determined by CellSearch. Whilst, the therapeutic efficacy of Clozotinib can be reliably responded by NanoVelcro assay and the changing of NanoVlecro CTC counts could accurately reflect disease progression. With CTCs capture and their corresponding ALK detection, dynamic monitoring of treatment effect for lung cancer patients could be realized during the process of Crizotinib treatment.

## Additional file

**Additional file 1.** Supplementary data.
