# Supplementary material for: Three-dimensional nanostructured substrates enable dynamic detection of ALK-rearrangement in circulating tumor cells from treatment-naive patients with stage III/IV lung adenocarcinoma
Source: J Transl Med. 2019 Jan 18;17:32. doi: 10.1186/s12967-019-1779-5 (PMC6339314; doi:10.1186/s12967-019-1779-5)
Supplement: Supplementary file 1 — Additional file 1. Supplementary data. [file 12967_2019_1779_MOESM1_ESM.doc]

**Three-dimensional nanostructured substrates enable dynamic detection of ALK-rearrangement in circulating tumor cells from treatment-naive patients with stage III/IV lung adenocarcinoma**

**1. CTC Enumeration studies**

According to a standard protocol, all blood specimens were collected into EDTA-contained vacutainer or CellSave® tubes and were processed within 24 h. For experiments using lysed blood, NH4Cl was added to whole blood in 10:1 v/v ratio and mixed for 20 min at room temperature. After centrifugation at 200 g for 5 minutes, the supernatant was removed and the cell pellet was re-suspended in an equivalent volume of buffer for capture experiments.、

**2. Fluorescent In Situ Hybridization Assay of Enriched CTC on SiNW**

SiNW chips were incubated for 2 hours at room temperature in a solution of methanol/acetic acid (9 to 1) for optimized fixation and then digested with pepsin solution. Pepsin digestion conditions on chips were specifically established for the Vysis LSI Dual Color ALK Break Apart Rearrangement Probe Kit (37°C, pepsin (Sigma, USA) at 10% in an HCL 0.01N). After washed with PBS 1X, chips were fixed at room temperature in a fixative solution containing formaldehyde (Sigma, USA) and dehydrated using successive baths containing increasing concentrations of ethanol. Hybridization process was optimized for ALK Probe. SiNW chips were co-denaturated then hybridized in a dark humid chamber overnight at 44°C. After incubation, filters were washed in Stringent Wash Buffer 1X (Dako) and then with Wash Buffer 1X (Dako) and dehydrated again in successive ethanol solutions. Filters were finally mounted using DAPI Vectashield (Dako).

**3. Immunohistochemical staining**

Formalin-fixed, paraffin-embedded tissue sections (4 μm) from NSCLC patients were made by rotary microtome (Leica, Wetzlar, Germany). All the slices were labeled with anti-TTF-1 (Thyroid transcription factor 1), anti-CEA (carcinoembryonic antigen), anti-CK-7(cytokeratin-7) and p63 (Dako, Glostrup, Denmark). 3,3′-Diaminobenzidine tetrahydrochloride was used to visualize the staining reaction, and Mayer hematoxylin was counterstained subsequently. To monitor batch consistency of all the staining slices, we used the known positive control and the negative control (an antibody dilution solution to take the place of primary antibody).
